# Shear Mechanism of a Novel SFCBs-Reinforced Composite Shear Connector: Experimental, Theoretical Investigations and Numerical Model

**DOI:** 10.3390/ma17143508

**Published:** 2024-07-15

**Authors:** Chengfeng Xue, Hao Huang, Qing Jia

**Affiliations:** 1 College of Civil Engineering, Xi’jing University, Xi’an 710123, China; xuechengfeng@xijing.edu.cn (C.X.); huanghao@stu.xijing.edu.cn (H.H.); 2 School of Highway, Chang’an University, Xi’an 710064, China

**Keywords:** steel-FRP composite bar, composite shear connector, push-out test, finite element analysis model, shear capacity prediction

## Abstract

Traditional stud and perfobond leiste (PBL) shear connectors are commonly used as load-transferring components in steel-concrete composite structures. Composite shear connectors fully utilize the advantages of traditional stud and PBL shear connectors. In order to maximize the advantages of composite shear connectors, a novel shear connector for complex environments was proposed. The steel-FRP composite bars (SFCBs) with excellent fatigue resistance and corrosion resistance were introduced to replace the steel bars. This study discussed the failure modes, load–slip curves, and load–strain curves of the composite shear connector. In addition, a finite element analysis (FEA) model was developed to analyze the influence of various factors on its shear behavior. Results showed that compared with traditional composite shear connectors, the introduction of SFCB resulted in a promotion of 7.85% in shear stiffness, and it also led to a significant increase of 63.61% in ductility, further enhancing the mechanical performance. Meanwhile, FEA models were well fitted to the test results, and parametric analysis showed variate effects on shear bearing capacity. In the end, an equation was established to calculate the shear capacity of composite shear connectors, which could provide a reference for further research and engineering applications.

## 1. Introduction

Composite structures are extensively employed in buildings, bridges, and other infrastructures, for the combination of steel and concrete is high performance with lightweight and efficient fatigue resistance [1,2]. The shear connector component takes an essential role in transmitting the interfacial shear force between two distinct materials and ensuring they work together as a structural unit [3,4,5]. In the practical design of shear connectors, it is critical to choose an efficient type of shear connectors in the complex environment and the verification of its different limit states requires a clear understanding of its load-carrying mechanisms.

**Figure 1:**
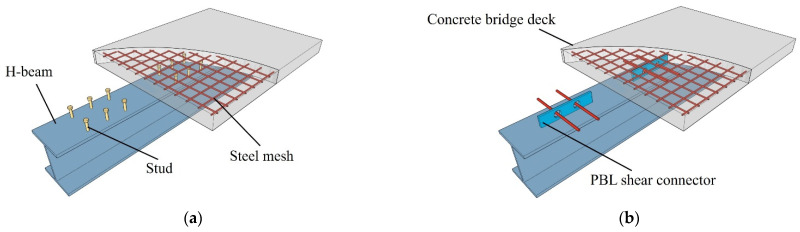
Shear connectors. (**a**) Composite beam with stud shear connectors; (**b**) composite beam with PBL shear connectors.

There are two often-used types of shear connectors, namely the stud shear connector and perfobond leiste (PBL) shear connector, which serve as flexible and rigid connectors respectively. As presented in Figure 1a, the stud shear connector offers a fast and efficient method through welding the studs onto the main girders in the construction stage.

These studs are then embedded into concrete, creating a strong and durable connection. Nevertheless, the strength of each stud is minimal, often requiring a dense arrangement of studs, which is not convenient for construction [6,7]. Zou et al. [8] suggested a novel prefabricated shear connector that leveraged the stud’s mechanical property, reducing the construction difficulty. Nguyen et al. [9] used stud connectors of large diameters and conducted experiments. The results showed that large-diameter studs could significantly improve shear capacity, but they also increased the risk of premature cracking of the concrete slab. Wang et al. [10,11] believed that using UHPC allowed for larger stud diameters, which avoided premature cracking of the concrete slab and increased the shear capacity of the shear connector. Another typical connector is the PBL shear connector. Figure 1b shows that this type of connector consists of the perforated steel plate, concrete dowel, and steel bar penetrating through the hole in the steel plate. Although it has significant advantages in construction, the high shear stiffness and limited deformation capability can result in stress concentration during both construction and use [12,13,14,15,16]. Liu et al. [17] used rubber flexible material as an inner lining within perforated steel plates, increasing the contact surface area for concrete within the holes. This solution helped prevent the steel plate from yielding deformation caused by stress concentration at the holes, but it also reduced the overall shear stiffness of the structure. Ma et al. [18] found that increasing the diameter of the hole resulted in an improvement in shear capacity. However, due to the limited height of the web plate, the structural ductility was reduced. Kim et al. [19,20,21,22] suggested a Y-shaped connector with a larger load-bearing area to enhance shear capacity, but it led to stress concentration at the root of the bent web plate.

A number of scholars have innovatively proposed many composite shear connectors. Gao et al. [23] studied an innovative prefabricated composite shear stud (PCSS), and its shear-slip behavior in composite bridges was investigated. A total of 12 specimens were fabricated, and push-out tests were carried out, along with finite element analysis. According to the result, the ultimate strength of PCSS had been increased by 39.1–67.3%. At the same time, the lateral restraint of the vertical steel plate was the key factor in improving the ductility of the specimen. Ma et al. [24] introduced a UHPC rubber sleeve stud connector consisting of an ordinary stud (OS) connector, a rubber sleeve, and a filled UHPC. Push-out tests and numerical simulations were carried out to study the shear performance and deformation capacity of the novel composite shear connector. Pardeshi et al. [25] introduced an innovative concave-type shear connector. Nonlinear Finite Element Analysis of Push-Pull Tests of shear connectors was conducted by ABAQUS (version 2022). The results showed that the stiffness of the concave-type shear connector was increased by a factor of 2.5, while the material usage was reduced by 20% under the same shear strength. Ma et al. [18] proposed T-Perfobond shear connectors. The results of the experiments and numerical simulations showed that specimen damage was divided into three stages: bond damage, concrete dowel breakage, and end concrete crushing. The hole diameter of the web plate reduced the strength and decreased the stability of the T-Perfobond shear connectors.

While steel-concrete composite bridges have excellent structural performance, the rapid deterioration of bridges under severe environmental conditions may lead to structural distress, even catastrophic failures [26,27,28,29,30]. Scholars have proposed using fiber-reinforced polymer (FRP) bars to improve the deteriorating structural performance [31,32,33,34,35]. FRP bars exhibit excellent tension performance. However, its low elastic modulus and poor deformability limit its application in structures [36,37,38]. Therefore, scholars have proposed a composite bar to share the advantages of the FRP bar and steel bar, a steel-FRP Composite Bar (SFCB).

Sun et al. [39] carried out a study on the mechanical properties of steel-FRP composite bars under tensile and compressive loads. SFCB, under compressive loading, had three failure modes. The ultimate strength of SFCB decreased slightly with increasing FRP content, compared to pure FRP bar. Wu et al. [40,41] Introduced a newly developed steel-fiber-reinforced polymer (FRP) composite bar (SFCB). Uniaxial tensile tests and cyclic tensile tests were conducted to study the mechanical properties of SFCB. The test results showed that the stress–strain curve of SFCB was a bilinear model, and the predicted results were in good agreement with the experimental results. Zhao et al. [42] introduced production techniques of the newly-developed SFCB with a round steel bar inner core and studied its mechanical properties and bonding properties with concrete. The experimental results demonstrated that the tensile performances of new types of SFCBs were better than conventional SFCBs. CFRP materials have excellent corrosion resistance. Zhou et al. [43] proposed the impressed current cathodic protection anode method to further enhance the corrosion resistance of SFCB bars. The system has great potential for application in marine concrete structures.

**Figure 2:**
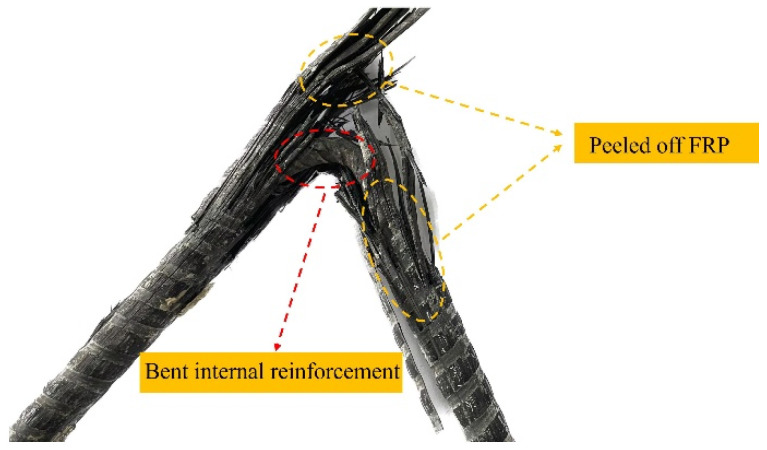
Destroyed SFCB.

As shown in Figure 2, the form of the SFCB after destruction has been displayed. The SFCB is a composite material formed by wrapping carbon fiber-reinforced polymer (CFRP) around the surface of the steel bar through a pultrusion process, which possesses a high elastic modulus and ultimate strength while exhibiting excellent ductility [44,45,46].

**Figure 3:**
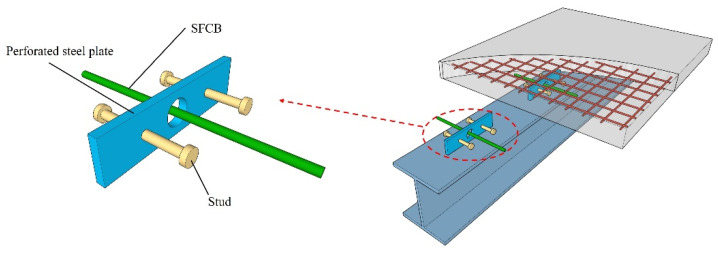
The novel composite shear connector.

Although researchers have conducted many studies to solve the problems of traditional connectors, further research is required. (1) Existing shear connectors face difficulty simultaneously possessing high shear stiffness and ductility, and the uneven internal stress distribution makes the concrete slab prone to brittle failure. (2) Currently, research on SFCB primarily focuses on tensile and flexural performance, with few studies on its shear performance. (3) While SFCB has excellent mechanical properties, its application in shear connectors needs to be further developed to promote the wide use of SFCB. In order to tackle these concerns, this study proposed a composite shear connector that combined the advantages of stud and PBL connectors. As shown in Figure 3, studs were horizontally arranged on perforated steel plates, and this arrangement could improve the stress distribution at the root of the studs, avoiding stress concentration and reducing the studs’ grouping effect. To further improve the structural ductility, SFCB was used as the penetrating rebar.

In the present study, the shear behavior of SFCB-reinforced composite shear connectors was investigated. The effect of penetrating rebar type was considered in the experimental program. The test results were analyzed to characterize the failure modes, load–slip curves, load–strain curves, and influence laws of the use of SFCB. Based on the test result, a finite element model was established, and the effect of hole number, stud length, hole diameter, and CFRP thickness ratio on the shear capacity was analyzed. The purpose of this paper is to introduce SFCB into composite shear connectors in order to propose a new type of composite shear connectors that can be applied in fatigue and corrosion environments, thus providing a certain degree of protection for composite structures in both of these complex environments.

## 2. Experiment Program

### 2.1. Test Specimens

**Figure 4:**
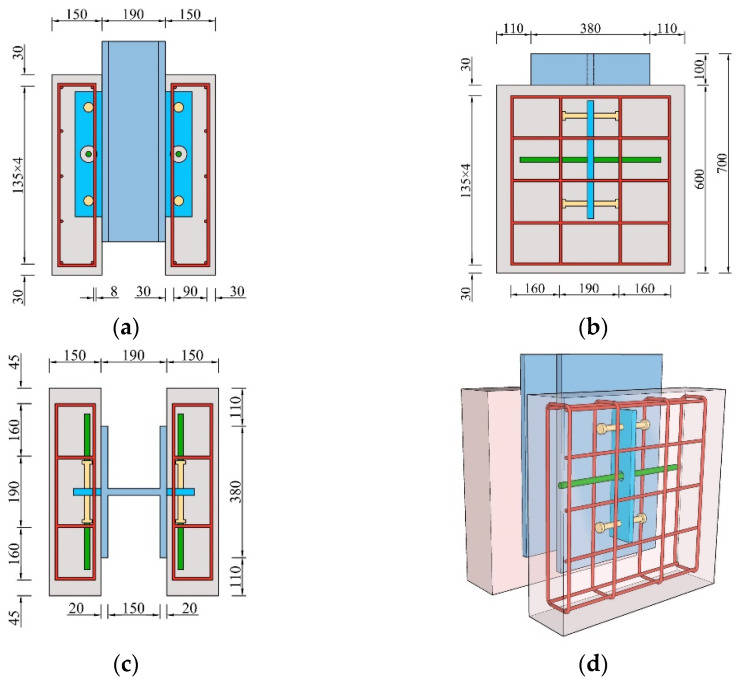
Dimension of test specimens (unit: mm). (**a**) Front view; (**b**) side view; (**c**) plan view; (**d**) overall dimension.

In this study, two specimens were designed and fabricated, in line with Eurocode 4 [47], to evaluate the mechanical behavior of SFCBs-reinforced composite shear connectors. The specific dimensions are shown in Figure 4 and Table 1.

The overall dimension of the specimen was 600 mm × 490 mm × 600 mm, and the size of the concrete slab was 600 mm × 600 mm × 150 mm. The section size of the H beam was 600 mm × 490 mm × 600 mm, and the thickness of the flange plate was 20 mm. The perforated steel plate, divided into a single hole, measured 375 mm × 80 mm × 16 mm. The stud was ML5AL type, with a diameter of 16 mm and a height of 80 mm. The stirrup’s diameter was 8 mm, both horizontally and vertically. The penetrating rebar was divided into the steel bar and SFCB, which had a diameter of 14 mm and a length of 450 mm. The SFCB consisted of a 7 mm ribbed steel bar encased in 7 mm CFRP, with a wrap in 35 turns. The rules for naming specimens are as follows: the letter “C” represents the composite shear connector, and “B” represents the application of SFCB as penetrating rebar. The first number represents the number of penetrating rebar, while the second number represents the rows of studs.

### 2.2. Materials

The concrete slab was made of C50-grade concrete, and the target strength was 50 MPa. Due to errors in the concrete pouring and curing process, the actual strength parameters of concrete need to be obtained through material testing on concrete specimens poured simultaneously. Following the 28-day standard curing period, the specimens undergo cube compression tests, elastic modulus tests, and cylinder compression tests to ascertain material parameters. Table 2 lists the mechanical parameters of the concrete.

The H-beam utilized Q355 steel, while the steel bar and stirrup utilized HRB400 steel. The parameters for SFCB were an inner rebar diameter of 7 mm, wrapped in 7 mm thick CFRP. The stud was ML15AL type with Q235 steel. The mechanical parameters of the steel are listed in Table 3.

We used elongation as the indicator of the ductility of the steel of the connectors and SFCB bars. The steel elongation we obtained was 20%. In determining the elongation of the SFCB bar, it was divided into CFRP and Steel bar, and the results obtained were 1.6% and 14.5%, respectively. Due to CFRP’s smaller elastic modulus than the steel bar, the inner rebar in the SFCB yields before CFRP fiber fracture when subjected to tension. The diameter of the inner rebar in SFCB is smaller than that of the steel bar, leading to a lower yield strength for SFCB. After the inner bar yields, the wrapped fiber continues to bear the tension force, and the excellent tensile strength of CFRP provides SFCB with a higher ultimate tensile strength. The SFCB exhibits a significant increase in stiffness after yielding.

### 2.3. Specimen Fabrication

**Figure 5:**
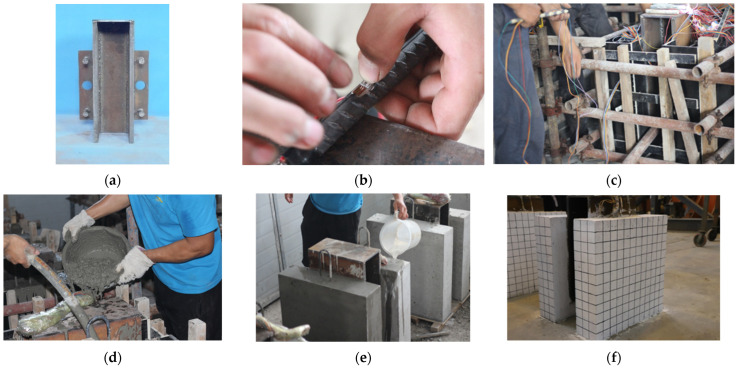
Specimen fabrication. (**a**) H-beam processed; (**b**) attach strain gauge; (**c**) template assembly; (**d**) concrete pouring; (**e**) standard curing of concrete; (**f**) specimen surface treatment.

Initially, the H-beam was processed, and reinforcing bars were spot-welded between the two flanges for support. Upon forming the specimen, these supporting bars were severed and removed. In order to mitigate the effects of eccentric loading, a flat steel plate was welded to the compression surface of the H-beam. Once the steel plate welding was complete, welding of the studs and perforated steel plates followed. Positions for the stud shear connectors were preliminarily marked with chalk to ease subsequent welding. The following steps involve attaching strain gauges, assembling templates, and placing penetrating rebars. The integrity of the strain gauges was subsequently tested. The contact surface was coated with oil upon final assembly to prevent chemical bonding. Following two days of concrete pouring, the wooden templates were removed, and a standard 28-day curing process was implemented. After the curing, the specimen’s surface was coated with a layer of white paint, and a 50 × 50 mm square grid was drawn using a marker pen. The specimen fabrication was thus completed. Figure 5 shows details of specimen fabrication.

### 2.4. Test Loading

**Figure 6:**
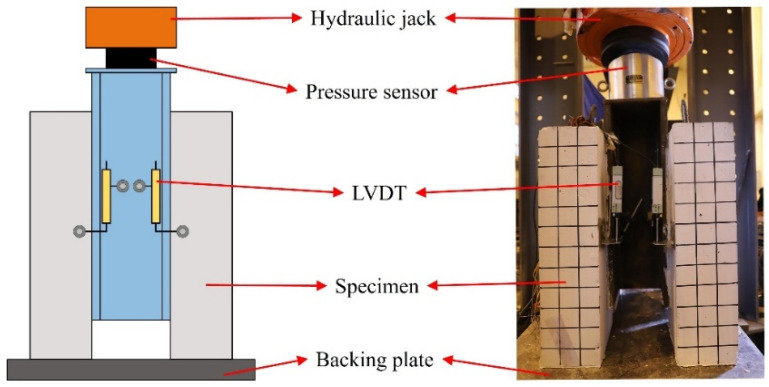
Loading device and layout of LVDTs.

As shown in Figure 6, a 5000 kN hydraulic jack was utilized.

In order to prevent eccentric loading throughout the test, the specimen bottom was uniformly covered with fine sand. Before loading, preloading was conducted as necessary to eliminate minuscule gaps and verify the sensor’s operational status. The formal loading method referred to specification GB/T 50152-2012 [48] and was divided into two loading stages. In the first stage, the loading rate was 0.3 kN/s until reaching the ultimate bearing capacity. In the second stage, displacement loading was applied at a rate of 0.02 mm/s. The loading process was considered complete whenever the slip value reached 40 mm, or the remaining bearing capacity decreased to less than 40% of the maximum bearing capacity.

**Figure 7:**
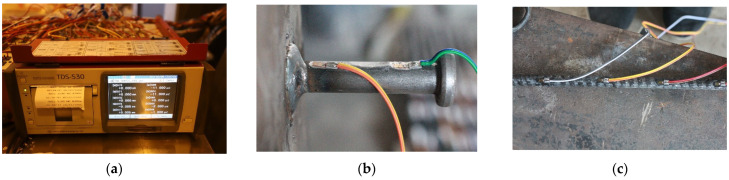
Specimen data collection. (**a**) TDS-530; (**b**) Strain gauge of stud; (**c**) Strain gauge of penetrating rebar.

During the loading process, the relative slip between the H-beam and the concrete slab was measured using four linear variable differential transformers (LVDTs), with the average value being recorded. Figure 7 shows the layout of the LVDT. The force sensor collected load data of the specimen, and a TDS-530 acquisition box gathered data every 3 s.

Embedded strain gauges were used to measure the strain of the penetrating rebar and stud. Relevant instruments and equipment are shown in Figure 7.

## 3. Experimental Result

### 3.1. Failure Modes

Firstly, we make the following assumptions about damage and force transfer in composite shear connectors:

(1) The symmetrically arranged shear connectors transmit shear forces of equal magnitude, and the structure is deemed to fail when one of them breaks;
(2) Shear forces and bending moments transmitted by studs and penetration bars are equal in magnitude.

**Figure 8:**
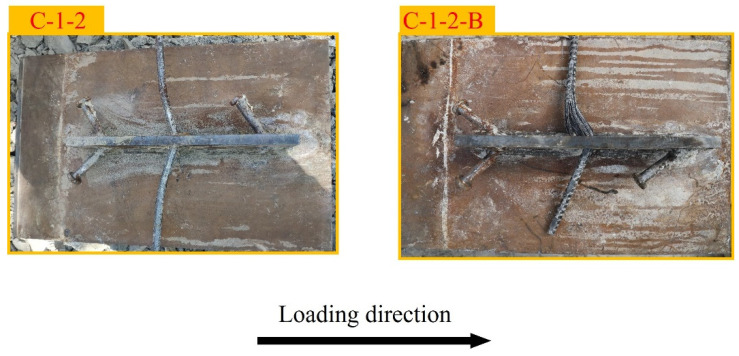
Failure modes of two specimens.

The failure modes of the two specimens are shown in Figure 8. The penetrating rebar passing through the perforated plate exhibited bending with varying angles.

**Figure 9:**
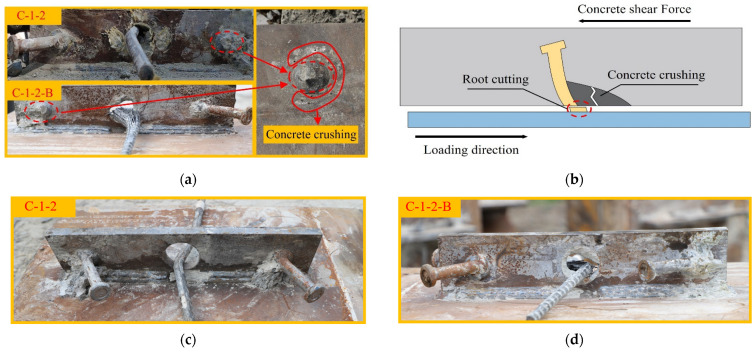
Shear mechanism of studs. (**a**) Failure mode of the studs; (**b**) stud failure mode diagram; (**c**) studs on the other side of C-1-2; (**d**) studs on the other side of C-1-2-B.

**Figure 10:**
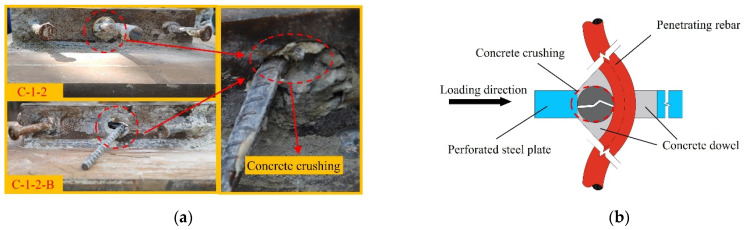
Shear mechanism of concrete dowels. (**a**) Failure mode of the concrete dowel; (**b**) concrete dowel failure mode diagram.

**Figure 11:**
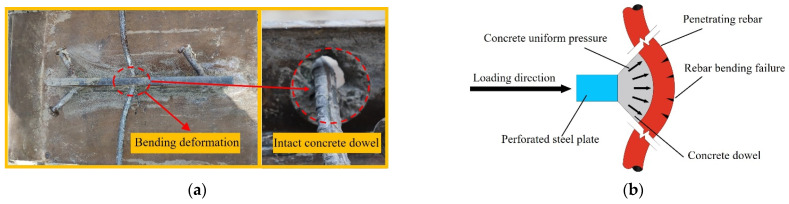
Shear mechanism of steel bar. (**a**) Failure mode of the steel bar; (**b**) steel bar failure mode diagram.

According to the appearance of the shear connector after failure, the failure modes of two specimens are classified into the following four types:

1. Stud failure mode: Figure 9a shows the failure mode of the studs. This failure mode mainly occurred at the stud root, and when the failure happened, the stud was first sheared off. As shown in Figure 9b, since the longitudinal stiffness of the concrete was high, the stud embedded in the bulk concrete was relatively vulnerable under the interfacial shear action. When reaching the ultimate failure load, a significant plastic slip occurred at the root of the stud, and the stud root was severed, resulting in the concrete at the stud root being crushed. By observing Figure 9c,d, we can find that studs on the other side of C-1-2 and C-1-2-B were severely deformed but not sheared off.
2. Concrete dowel failure mode: This failure mode mainly occurred in the concrete dowels, and when the failure happened, the concrete dowels were crushed. As shown in Figure 10a, in the case of concrete dowel failure mode, the steel bars underwent substantial bending deformation after yielding. Figure 10b shows that the concrete dowel was a force transmission medium between the steel bar and perforated steel plate, bearing the load until it reached the strength limit. The concrete dowel failure could easily lead to a rapid development of internal cracks in the concrete slab, forming a continuous surface, which could result in the overall failure of the structure.
3. Steel bar failure mode: This failure mode mainly occurred at the steel bar, and the concrete dowels remained intact. Figure 11a shows the steel bar failure mode. The horizontal arrangement of the stud ensures a more uniform distribution of the stress within the PBL shear connector, and both the perforated steel plate and the concrete dowel retained their integrity. As shown in Figure 11b, The concrete dowel gradually diffused and transferred the shear force to the steel bar. Owing to the compression force from the concrete dowel, the force state in the steel bar transformed from shear to shear-bending. The outer side of the steel bar experienced significant plastic deformation, and the final failure mode was a shear-bending failure.

**Figure 12:**
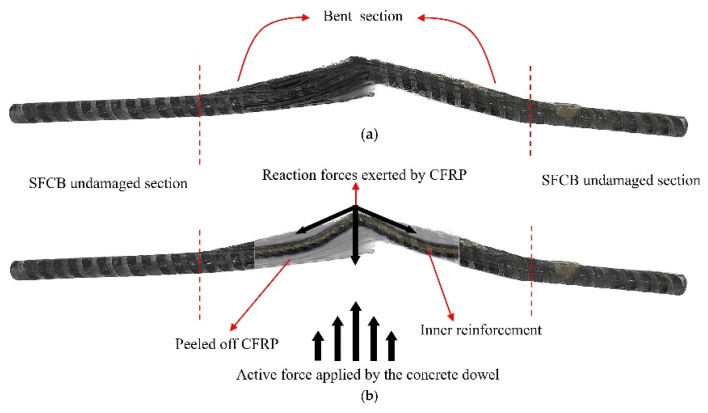
SFCB failure mode. (**a**) Appearance of SFCB; (**b**) SFCB stress transfer mechanism.

SFCB failure mode: This failure mode mainly occurred at the SFCB, and when the failure happened, the outer fibers of the SFCB were damaged, while the concrete dowel remained intact. As shown in Figure 12a, SFCB underwent bending under the action of the steel plate and concrete dowel.

Outside these bent sections, CFRP wrapped tightly around the internal reinforcement ensures synergy between CFRP and reinforcement. As shown in Figure 12b, the concave side CFRP was gradually peeled off from the internal steel with the deformation of the reinforcement within the bent sections. Gaps between the reinforcement and the CFRP led to drastic changes in tension on the CFRP and eventual shearing. Shear damage of CFRP on the concave side occurred under the action of the concrete dowel in the bending section. At this point, the SFCB failed for the first time in terms of stiffness. Internal reinforcement came to yield by bending moment, with the load increased. At the same time, the SFCB secondary stiffness failed.

**Figure 13:**
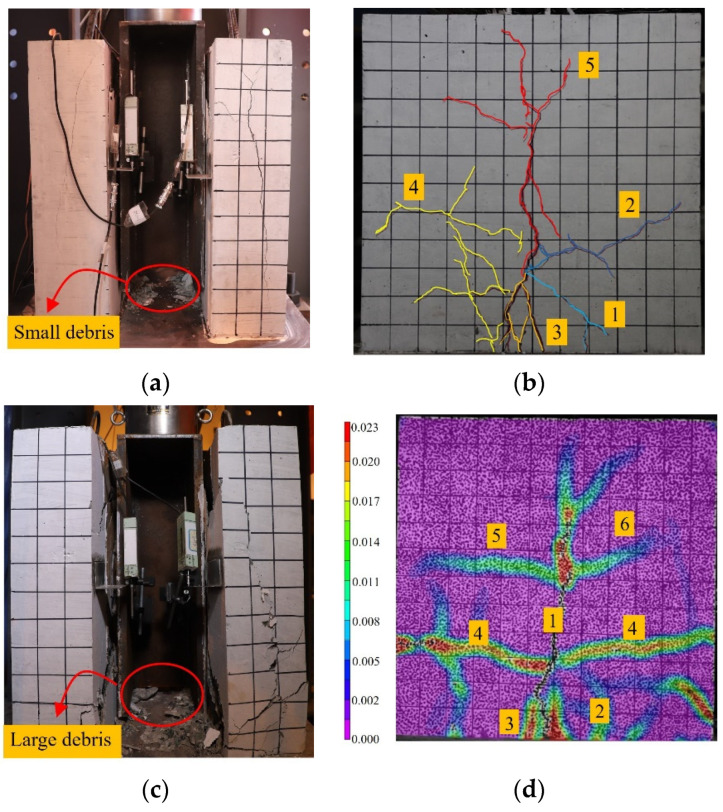
Deformation state of the concrete slab after failure. (**a**) C-1-2 front view; (**b**) C-1-2 side view; (**c**) C-1-2-B front view; (**d**) C-1-2-B side view.

The failure modes of the concrete slab can be categorized into splitting failure and crushing failure, with the former shown in Figure 13b.

No significant phenomena were observed on the concrete slab of C-1-2 during the initial loading stage. However, at $0.79P_{u}$ (ultimate bearing capacity), a small amount of concrete debris fell. Upon further loading to $0.87P_{u}$, a vertical crack (Crack 1) appeared on the concrete slab, progressing from the bottom towards the upper left. At $0.98P_{u}$, cracks 2, 3, and 4 suddenly emerged, converging with Crack 1 to form the main vertical crack (Crack 5). This phenomenon marked the onset of the load drop phase, during which the cracks gradually widened and were accompanied by a small amount of falling concrete debris.

The crushing failure mode is shown in Figure 13d. Digital image correlation (DIC) technology was utilized to depict the crack propagation of C-1-2-B. At $0.85P_{u}$, cracks 1, 2, 3, and 4 successively appeared, each progressing in its direction. Upon loading to $P_{u}$, the slip value rapidly increased, marking the onset of the load drop phase. When the load decreased to $0.87P_{u}$, cracks 5 and 6 emerged, ultimately forming a main crack and causing a significant amount of concrete debris to fall.

Traditional shear connectors frequently encountered uneven stress distribution, leading to local failure and subsequent total collapse. In contrast, composite shear connectors prevented concrete from crushing at the hole of the perforated steel plate, thus significantly improving the unevenness of deformation compared with traditional shear connectors. Compared with C-1-2, C-1-2-B exhibited more extensive transverse crack development at the penetrating rebar site, and the overall distribution of cracks in the structure was more uniform than C-1-2. This phenomenon was because SFCB possesses characteristics of high ductility and “secondary stiffness”. Significant deformation resulted in the redistribution of internal stress within the specimen, ensuring that each component was adequately stressed.

### 3.2. Load–Slip Curves

The mechanical properties of composite shear connectors were investigated by analyzing the data collected by the sensors. Table 4 lists the test results of two specimens.

It was found that the mechanical performance of the SFCBs-reinforced composite shear connectors exhibited a similar shear capacity when compared with traditional composite shear connectors. However, SFCB in composite shear connectors improved shear ductility and increased the structures’ stiffness.

**Figure 14:**
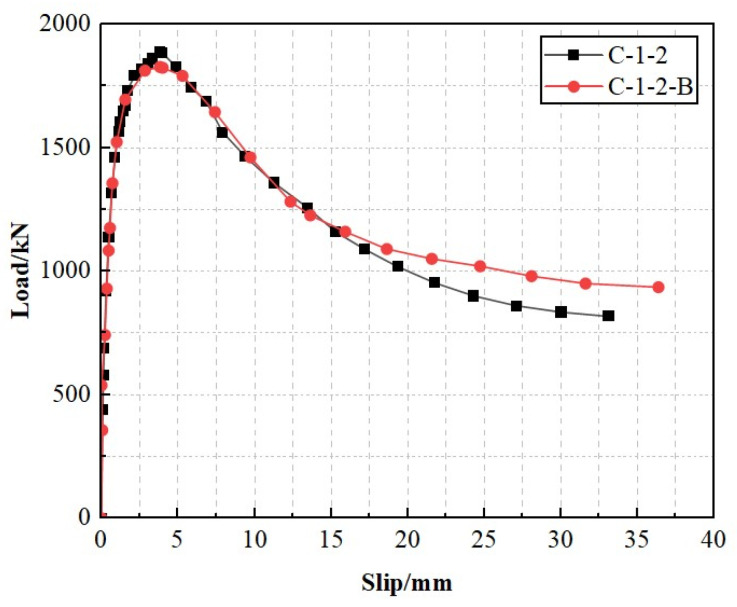
Load–slip curves.

Figure 14 shows the load–slip curve of composite shear connectors, which go through three stages: the elastic stage, plastic stage, and descending failure stage.

The load–slip curve exhibited relatively large slopes when two composite shear connector specimens were within the elastic phase. The slip values at this stage were approximately 0–1.0 mm, corresponding to a load of approximately $0.8P_{u}$. The slopes of the load–slip curve gradually decreased, showing nonlinear growth. The slip values were approximately 1.0–3.8 mm, with the corresponding load at this stage being $P_{u}$. During the descending failure stage, the studs and the penetrating rebar experienced significant plastic deformation, resulting in a reduction in the shear capacity. This deformation also led to the fast propagation of cracks until the structural failure or slip reached a predefined limit value. Meanwhile, Figure 14 shows that the load–slip curve of composite shear connectors using SFCB was relatively smooth compared with those using steel bars. SFCB did not cause rapid stress variation when the inner rebar was in a yielding condition, thus avoiding continuous failure. Additionally, the shear capacity of SFCB-reinforced composite shear connectors was not significantly different from steel bar- reinforced composite shear connectors. However, when the slip exceeded 15 mm, the bearing capacity of SFCB-reinforced composite shear connectors was always greater than traditional composite shear connectors, indicating that the posterior stiffness of the novel composite shear connector was greater than that of the conventional composite shear connectors. These phenomena made using SFCB as the penetrating rebar in composite shear connectors feasible.

### 3.3. Load–Strain Curves

**Figure 15:**
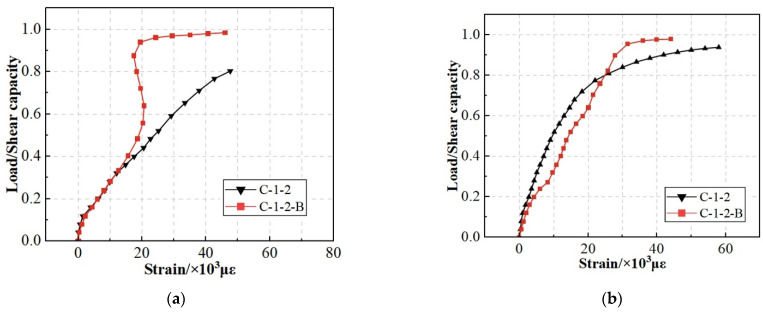
Load–strain curves of C-1-2 and C-1-2-B. (**a**) Load–strain curves of stud; (**b**) Load–strain curves of penetrating rebar.

Figure 15a shows the load–strain curve of the studs. The vertical axis indicates the ratio of the load to shear capacity.

Figure 15a shows that when the load reached $0.5P_{u}$, the C-1-2-B curve slope was consistently more significant than that of C-1-2. With the rapid increase in load, the strain exhibited a relatively slow rate of increase. This appearance was attributed to the reason that compared with steel bars, the use of SFCB resulted in a more uniform stress distribution. The stress that was initially borne by the studs was redistributed to other components, leading to a higher curve slope after the yield of the studs. Figure 15b shows the load–strain curve of the penetrating rebar. Figure 15b shows that the penetrating rebar of C-1-2-B yielded later than that of C-1-2. This appearance was attributed to the reason that the “secondary stiffness” provided by the CFRP layer reduced the strain growth rate after yielding the inner rebar.

### 3.4. Effect of the Type of Penetrating Rebar

**Figure 16:**
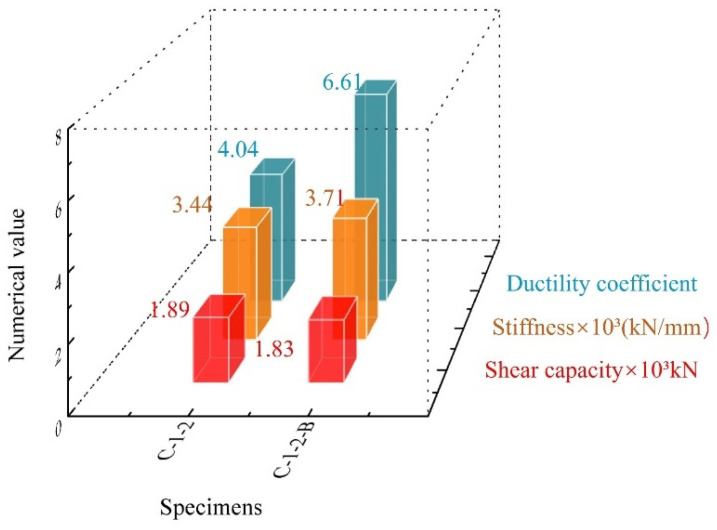
Influence of the type of penetrating rebar.

As shown in Figure 16, compared with steel bar specimens, the SFCB specimens exhibited a slight decline in shear capacity; however, a significant increase in shear stiffness and ductility of 7.85% and 63.61%, respectively.

The above results were mainly due to the following reasons: (1) Compared with the steel bar, SFCB had better ductility. The excellent tensile performance of CFRP provided larger deformation space for the inner rebar, improving the stress distribution in the connector and thus enhancing the ductility of the structure. (2) At the same time, the thickness of CFRP weakened the cross-sectional area of the steel bars, leading to a minor reduction in the shear capacity.

## 4. Finite Element Analysis and Validation

### 4.1. General

**Figure 17:**
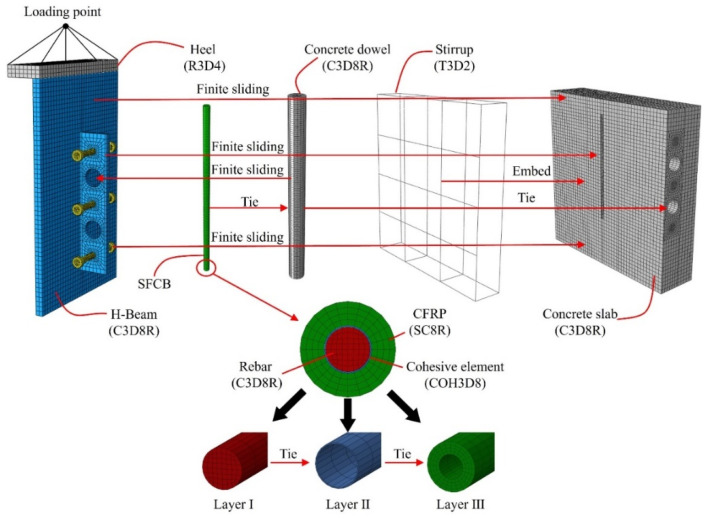
Models and meshes for various components.

In order to avoid inaccuracies in dynamic analysis, this section simulated the shear connectors using the ABAQUS standard analysis method [49]. As shown in Figure 17, the H beam, concrete slab, penetrating rebar, stud, and perforated steel plate were simulated using the C3D8R solid element.

To more accurately simulate the mechanical properties of SFCB, an SC8R continuous shell element was used to simulate the CFRP layer. The stirrups were simulated using the T3D2 truss element.

**Figure 18:**
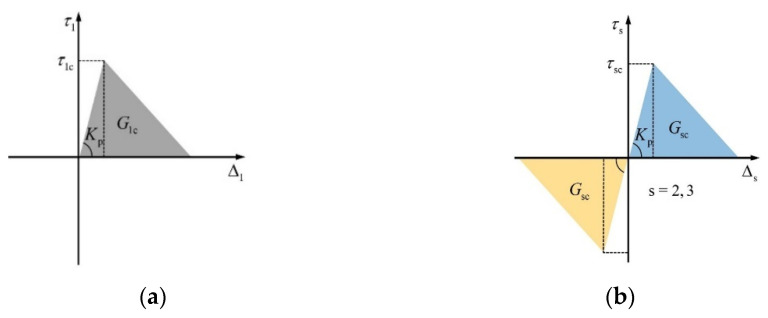
The traction separation law of cohesive element [50]. (**a**) Normal mode; (**b**) shear mode.

The cohesive element COH3D8 was utilized to simulate the bonding behavior between the CFRP and the inner rebar. As shown in Figure 18, a bilinear cohesive model was used to simulate the bonding behavior at the interface [50]. $\tau_{\mathrm{sc}}$ (S = 1, 2, 3) are the interface strength; $G_{\mathrm{sc}}$ (S = 1, 2, 3) are the interface fracture toughness; $\Delta_{s}$ (S = 1, 2, 3) is the separation vector at the interface; $K_{p}$ is the penalty stiffness.

Interface failure was simulated through normal mode and shear mode. In most cases, $G_{s2}$ in shear mode is considered to be the same as $G_{s3}$. The specific parameters of the cohesive elements are shown in Table 5 [50,51,52].

The stud, penetrating rebar, and concrete dowel were essential components, and the element size was set to 4 mm. The size of the remaining component element was set to 12 mm and locally densified, and the mesh was within the contact range of the studs and perforated plates. By dividing different sizes of meshes, the calculation time was reduced while also enhancing the accuracy of the calculations.

Figure 17 shows a half model, applying symmetry constraints to the model along the symmetry plane. The encastre constraints were applied to the underneath of the concrete slab. The studs, perforated steel plate, and H-beam were integrated into a single model, ensuring all contact surfaces shared nodes to enhance the precision of calculations.

Embedded constraints were used between the stirrup and the concrete slab. Meanwhile, tie constraints were applied between the concrete dowel and concrete slab and between the steel bar and concrete dowel. As shown in Figure 17, the SFCB was divided into three layers: Layer I, II, and III consisting of rebar, COH3D8 elements, and CFRP, respectively. Layer II had no actual thickness. Between the rebar and COH3D8 elements, as well as between the cohesive elements and CFRP, tie constraints were utilized. Similarly, a tie constraint was used between the CFRP and concrete dowel. The contact behavior for the rest of the contact surfaces was defined by finite sliding contact.

### 4.2. Material Properties in the Modelling

#### 4.2.1. Concrete

The behavior of concrete is approached with Concrete Damaged Plasticity (CDP) from the ABAQUS material library. The parameter values of the CDP model are shown in Table 6.

**Figure 19:**
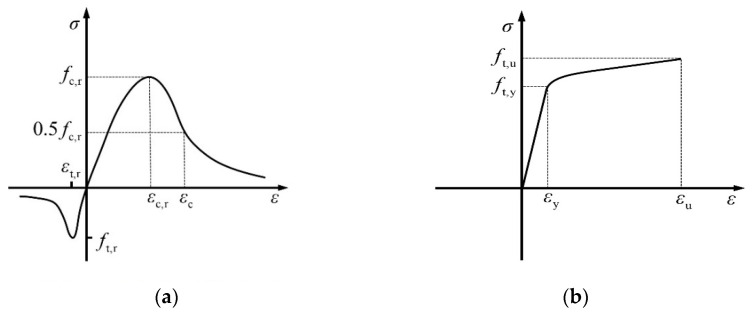
Stress–strain curves. (**a**) Stress–strain curve of concrete; (**b**) true stress–strain curve of steel.

Figure 19a shows the uniaxial stress–strain curve of concrete [55].

The compression segment of the curve can be plotted using Equations (1)–(5).

(1) $$\sigma =\left(1-d_{c}\right)E_{c}\varepsilon$$

(2) $$d_{c}=\left\{\begin{array}{cc} 1-\frac{\rho_{c}n}{n-1+x^{n}} & x\leq 1\\ 1-\frac{\rho_{c}}{\alpha_{c}{\left(x-1\right)}^{2}+x} & x>1 \end{array}\right.$$

(3) $$\rho_{c}=\frac{f_{c,r}}{E_{c}\varepsilon_{c,r}}$$

(4) $$n=\frac{E_{c}\varepsilon_{c,r}}{E_{c}\varepsilon_{c,r}-f_{c,r}}$$

(5) $$x=\frac{\varepsilon }{\varepsilon_{c,r}}$$

where $\alpha_{c}$ is a descending segment coefficient of the curve; $f_{c,r}$ is typical concrete uniaxial compressive strength; $\varepsilon_{c,r}$ is the concrete ultimate compressive strain; $d_{c}$ is a damage index for the compression behavior of concrete.

The tensile stress–strain curve can be plotted using Equations (6)–(9).

(6) $$\sigma =\left(1-d_{t}\right)E_{c}\varepsilon$$

(7) $$d_{t}=\left\{\begin{array}{ll} 1-\rho_{t}\left[1.2-0.2x^{5}\right] & x\leq 1\\ 1-\frac{\rho_{t}}{\alpha_{t}{\left(x-1\right)}^{1.7}+x} & x>1 \end{array}\right.$$

(8) $$x=\frac{\varepsilon }{\varepsilon_{t,r}}$$

(9) $$\rho_{t}=\frac{f_{t,r}}{E_{c}\varepsilon_{t,r}}$$

where $\alpha_{t}$ is a descending segment coefficient of the curve; $f_{t,r}$ is typical concrete uniaxial tensile strength; $\varepsilon_{t,r}$ is the concrete ultimate tensile strain; $d_{t}$ is a damage index for the tensile behavior of concrete.

#### 4.2.2. Steel and Reinforcing Steels

The engineering stress–strain curve of steel is determined through material testing, and the cross-sectional area of the steel is assumed to be a constant value during the testing process. Owing to load values decreasing after the steel yields due to necking, the engineering stress decreases. The true stress considers the necking effect of the steel, which increases as the cross-sectional area decreases after yielding [56,57]. To simulate the true stress–strain correlation of steel precisely, the constitutive relationship shown in Figure 19b was adopted to model the true stress–strain curve of steel [58]. The first phase was a linearly elastic stage, where the curve slope indicated the elastic modulus, symbolized as $E_{s}$. The second phase of the curve was the yield stage, during which the steel transitions into the hardening stage. In this stage, the steel continued to undergo stress and strain until it reached the peak stress, denoted as $f_{t,u}$ and the peak strain, denoted as $\varepsilon_{u}$. The true stress–strain curve can be plotted using Equations (10)–(12).

(10) $$\varepsilon =\frac{\sigma }{E_{s}}+0.02\left(\frac{\sigma }{f_{t,y}}\right)$$

(11) $$\varepsilon_{\mathrm{true}}=ln\left(1+\varepsilon \right)$$

(12) $$\sigma_{\mathrm{true}}=\sigma \left(1+\varepsilon \right)$$

#### 4.2.3. CFRP

The continuous shell element was used to simulate the mechanical behavior of CFRP. The number of ply layers in the thickness direction was 8. The damage behavior of CFRP was simulated using the Hashin damage criterion. Specific parameter values can be found in the literature [59,60,61], as shown in Table 7.

**Figure 20:**
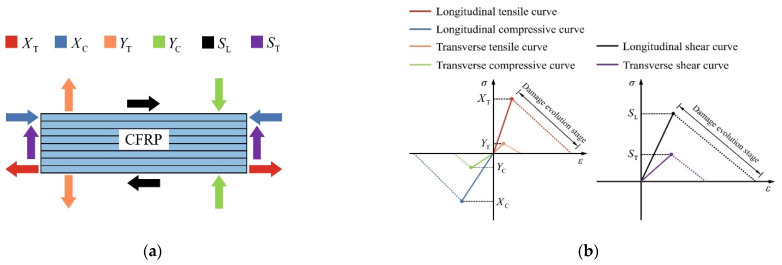
Hashin damage criterion. (**a**) Hashin parameters of CFRP; (**b**) stress–strain curves of CFRP.

The parameters of the CFRP material given in Table 7 were obtained through standard tests (ASTM-D4762, [62]). The values of fracture energy in Table 7 are extracted from the properties of similar materials [61]. Their research results proved that the simulation results closely matched the experimental values when the model used these CFRP material properties. The stress–strain curve of CFRP using Hashin damage is shown in Figure 20. Initially, the stress–strain curve of CFRP linearly increased until it reached the ultimate strength in the corresponding direction. After reaching the ultimate strength, the curve entered the damage evolution stage. The damage evolution behavior of CFRP was determined by fracture energy.

### 4.3. FEA Model Validation

**Figure 21:**
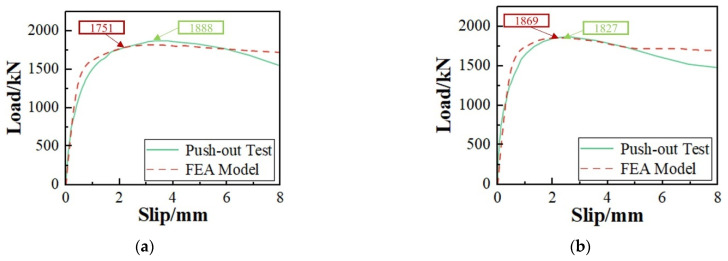
Load–slip curve comparison. (**a**) C-1-2; (**b**) C-1-2-B.

As shown in Figure 21, the FEA model accurately simulated the load–slip curves of specimens in the elastic, plastic, and descending failure stages. The CDP model allowed the concrete elements to bear loads even after failure [63]. During the failure stage, it was observed that the load–slip curve of the FEA model exhibited larger values than the experimental values due to this characteristic.

Due to excessive structural nonlinearity, the internal and external forces of the structure are highly unbalanced when a large number of concrete elements fail [64]. This resulted in an error between the FEA model and test results. Table 8 shows that the minimum error is 0.93% and the maximum error is 9.29%, both within 10%, showing that the numerical analysis results are accurate.

**Figure 22:**
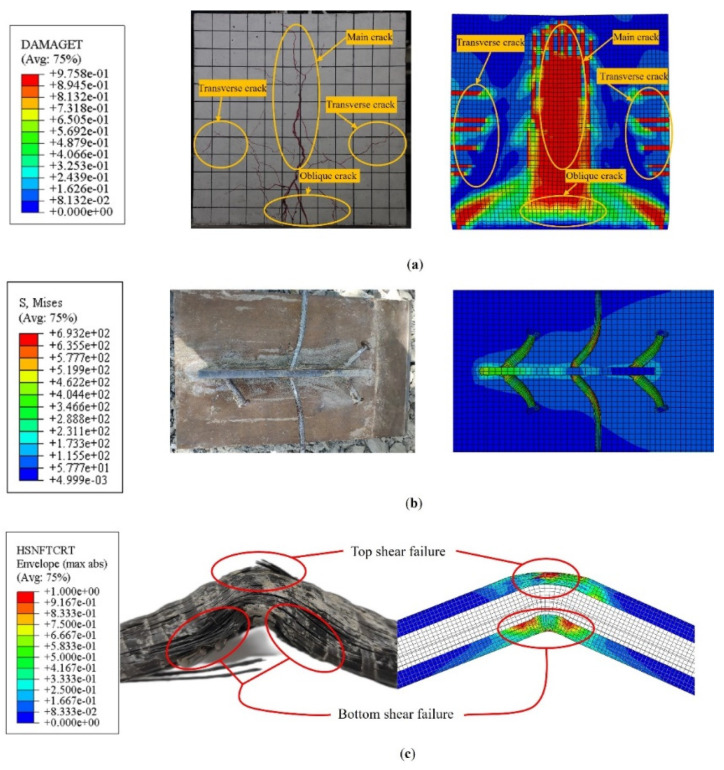
Failure modes comparison. (**a**) Concrete failure mode comparison between FEA model and experimental results; (**b**) shear connector failure mode comparison between FEA model and experimental results; (**c**) SFCB failure mode comparison between FEA model and experimental results.

Figure 22a shows concrete slab tensile damage distribution.

The FEA model precisely simulated the location of tensile damage in concrete, which corresponded closely to the actual cracking location in the concrete slab. The FEA model reproduced the crack propagation trend, with initial cracks appearing at the midline of the concrete slab and gradually extending outward, generating transverse and oblique cracks. The oblique cracks continuously developed upwards, ultimately forming the main crack. As Figure 22b shows, the FEA model accurately simulated the deformation of the studs and penetrating rebars. The load distribution of the composite shear connectors was uniform, and the inclination angles of its various components were broadly consistent. The distribution of damage in the SFCB fiber matrix is shown in Figure 22c, and the FEA model accurately simulated the failure modes of the CFRP layer. The top fiber was partially cut off after stretching, and the outermost fiber peeled off. The fibers on both sides were compressed and then partially cut off, and the outer layers of fibers were peeled off. The results indicate that the FEA model accurately simulated the load–slip curve, shear capacity, and structural failure mode of shear connectors, indicating the accuracy of the model. The results indicate that the FEA model accurately simulated the load–slip curve, shear capacity, and structural failure mode of shear connectors, indicating the accuracy of the model.

### 4.4. Parametric Analysis

The shear capacity is analyzed considering five factors: number of holes, stud length, hole diameter, and the CFRP thickness ratio to assess the influence of different parameters. The primary purpose of this section is to study the effects of those five factors. The following subsections describe the variations in shear capacity resulting from various factors.

#### 4.4.1. Effect of the Number of Holes

**Figure 23:**
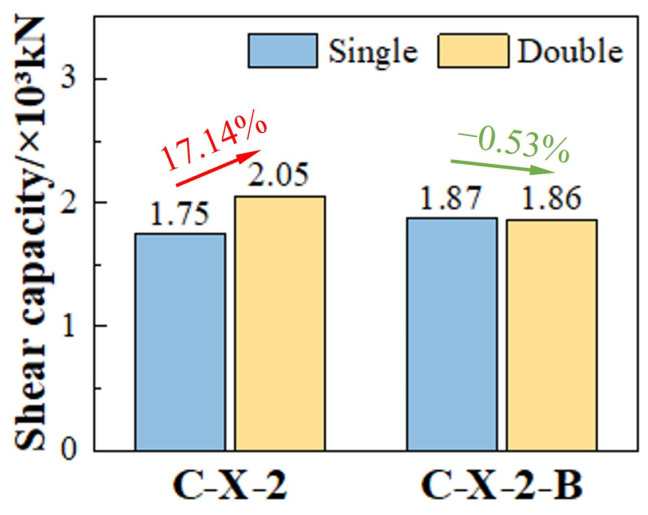
Influence of hole number on shear capacity.

As shown in Figure 23, in the FEA model, when the number of holes increased to two, the shear capacity of the SFCB-reinforced composite shear connectors exhibited a slight decline in shear capacity.

However, for conventional steel-reinforced composite shear connectors, increasing the number of holes can effectively improve the structural shear capacity. This phenomenon was mainly due to the following reasons: (1) For conventional steel-reinforced composite shear connectors, with the increase in the number of holes, the cross-sectional area of the penetrating rebars and concrete dowels also increased. (2) In the SFCB-reinforced composite shear connectors compared with single-hole connectors, the stress distribution in multi-hole connectors was uneven [3]. The initial low elastic modulus of SFCB caused some concrete dowels to fail before other parts reached the ultimate state; thus, the impact on the structural shear capacity was not significant.

#### 4.4.2. Effect of Stud Length

**Figure 24:**
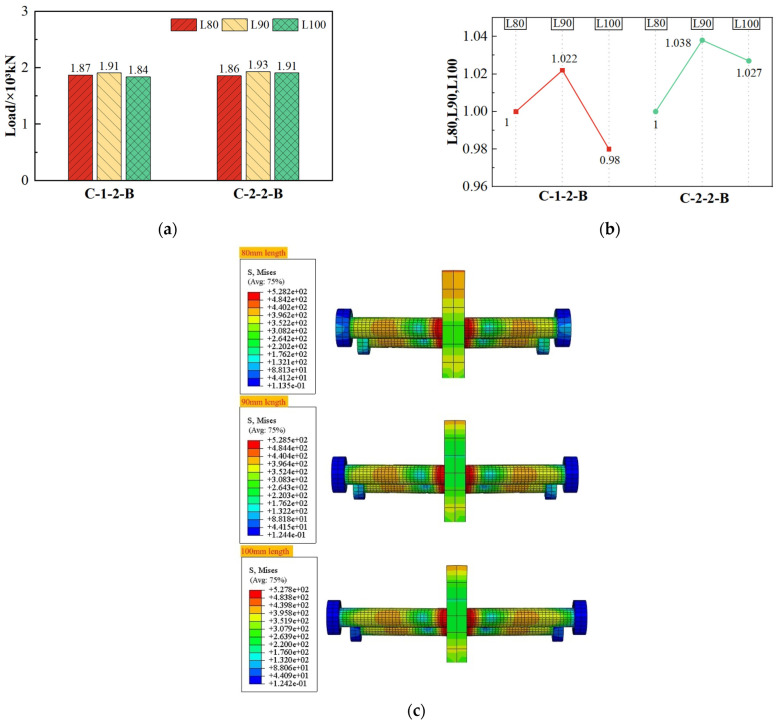
Effect of stud length on shear capacity. (**a**) Shear capacity; (**b**) comparison value of shear capacity; (**c**) stress distribution of studs under the same displacement load.

As shown in Figure 24, the change in shear capacity with the increase in stud length was relatively low, with a maximum change of 3.8%, indicating that the effect of stud length on the shear capacity of composite shear connectors was minor.

Furthermore, when the stud length increased from 90 mm to 100 mm, both C-1-2-B and C-2-2-B exhibited a decrease in their shear capacity. Combined with Figure 24c, it suggests that the stress areas on the studs showed no major changes, with the stud length increased. At the same time, the changes in the length of the studs have little effect on the load capacity due to the fact that the damage to the studs is mainly caused by the destruction of the roots. But compared with the specimen whose stud length increased to 90 mm and 100 mm, the stress cloud diagram of the prefabricated steel plate of the specimen with less stud length (80 mm) changes significantly. This is due to the fact that the longer the studs are, the more they weaken the stress of the prefabricated steel plate. Therefore, an excessive increase in stud length has the potential to accelerate structural damage.

#### 4.4.3. Effect of Hole Diameter

**Figure 25:**
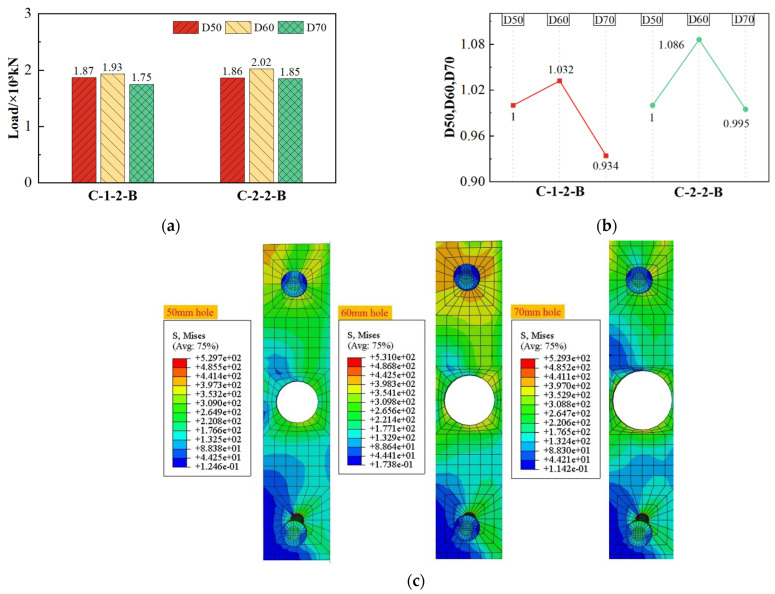
Effect of hole diameter on shear capacity. (**a**) Shear capacity; (**b**) comparison value of shear capacity; (**c**) stress distribution of perforated plate under the same displacement load.

As shown in Figure 25, when the diameter of the hole increased from the original 50 mm to 70 mm, changes in shear capacity showed a tendency to increase and then decrease, and the shear capacity increased by a minimum of 3.2% and decreased to a maximum decrease of 6.6%.

This result illustrates that appropriately increasing the hole diameter could enhance the shear capacity of the composite shear connector. However, an excessive increase in hole diameter may result in a reduction in shear capacity. Combined with Figure 25c, it reveals that an appropriate increase in the diameter of the hole makes the concrete force more uniform, and force on SFCB-concrete better transfers to the prefabricated steel plate. Thus, the shear capacity has been increased to a certain extent. However, when the diameter of the hole is raised to 70 mm, the shear capacity of the specimen decreases sharply. The web area of the prefabricated steel plate will be weakened as a result of an excessive increase in the diameter of the hole. At the same time, the larger diameter of the concrete dowel in the hole leads to earlier cracking of the SFCB-concrete dowel as a whole, which is not conducive to shear transfer. This phenomenon is similar to that in reinforced concrete structures, where excessive protective layer thickness may lead to premature cracking of the structure.

#### 4.4.4. Effect of CFRP Thickness Ratio

**Figure 26:**
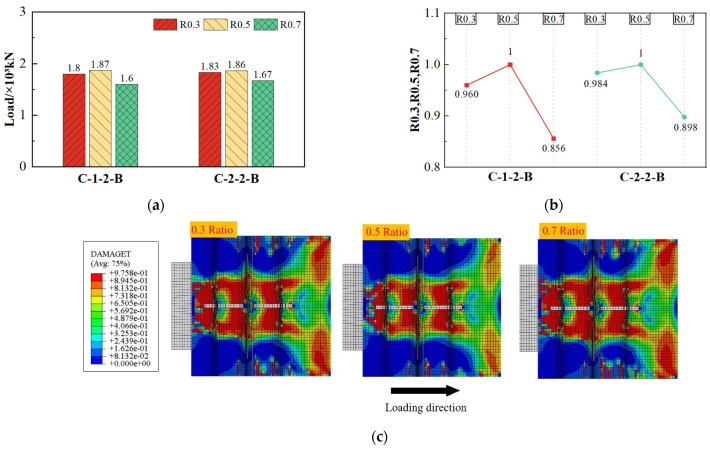
Effect of CFRP ratio on shear capacity. (**a**) Shear capacity; (**b**) comparison value of shear capacity; (**c**) concrete damage under the same displacement load.

As a composite reinforcing bar, the diameter of SFCB is determined by the summarization of the inner steel bar diameter and the outer CFRP fiber cladding. This means that even if the SFCB diameter is the same, different ratios of CFRP thickness could result in significant differences in its mechanical performance. As shown in Figure 26, when the ratio of CFRP thickness increased from 0.3 to 0.5, the change in shear capacity was minor, with a maximum increase of 4%.

However, when the thickness ratio further increased to 0.7, the shear capacity decreased to varying degrees, with a minimum decrease of 10.2%. Combined with Figure 26c, it is evident that when the thickness ratio increased from 0.3 to 0.7, the internal failure area of the composite shear connector did not differ significantly. Regardless, when the thickness ratio increased to 0.7, the failure area of the concrete dowel showed a significant increase. After analysis, it is believed that reducing the ratio of CFRP thickness made the SFCB behave more like a steel bar, resulting in a negligible impact on the shear capacity. The shear performance of CFRP is lower than that of steel bars. When the thickness ratio of CFRP became too large, it weakened the shear stiffness of the SFCB cross-section. At the same time, due to the slight deformation of the composite connectors in the elastic stage, the tensile performance of CFRP could not be fully utilized, thus reducing the shear capacity of the connectors.

## 5. Shear Capacity Prediction

**Figure 27:**
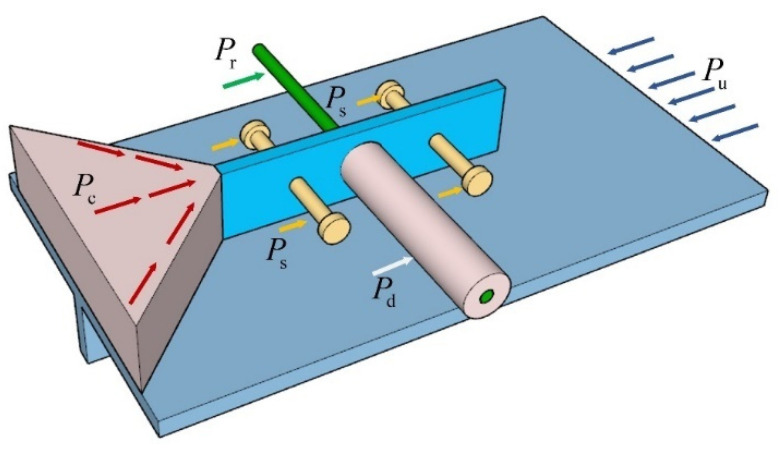
Load transfer diagram.

At the ultimate limit state, as shown in Figure 27, the applied force $P_{u}$ can be resisted by four parts, viz. the penetrating rebar ($P_{r}$), end-bearing concrete ($P_{c}$), concrete dowel ($P_{d}$), and stud ($P_{s}$).

Hence, the whole shear capacity of composite shear connectors ($P_{u}$) can be expressed as follows:

(13) $$P_{u}{=P}_{r}{+P}_{c}{+P}_{d}{+P}_{s}$$

The material strength primarily dictates the shear capacity of the penetrating rebar. When steel bar is used as the penetrating rebar, the steel bar undergoes shear force and bending moment during the loading process. The yield strength of the rebar has a direct impact on its shear capacity. When SFCB is used as the penetrating rebar, the structural shear capacity will vary slightly. Using the yield strength of penetrating rebar ($f_{\mathrm{yr}}$), introducing the partial factor ($k_{1}$) and the SFCB variation factor (μ). The Equation used to compute the effect of penetrating rebar can be expressed as follows:

(14) $$P_{r}=n_{1}\mu k_{1}A_{r}f_{\mathrm{yr}}$$

where $n_{1}$ is the number of holes; $A_{r}$ is the cross-sectional area of the penetrating rebar; and when the penetrating rebar is steel bar, μ=0.

The contact area between the concrete and the bottom of the perforated steel plate primarily determines the compressive strength of the end-bearing concrete. Introduce partial factor ($k_{2}$). The Equation used to compute the effect of end-bearing concrete can be expressed as follows:

(15) $$P_{c}=k_{2}A_{c}f_{c}$$

where $A_{c}$ is the bottom area of the perforated steel plate; $f_{c}$ is the compressive strength of the concrete.

Compared with compressive strength, concrete exhibits a lower shear strength. However, The concrete strength will be improved due to the three-axis compression through the penetrating rebar, surrounding concrete, and perforated steel plate [65]. Introduce partial factor ($k_{3}$). The Equation used to compute the effect of concrete dowel can be expressed as follows:

(16) $$P_{d}=n_{1}k_{3}A_{d}{f_{c}}^{0.57}$$

where $A_{d}$ is the cross-sectional area of the concrete dowel.

As composite shear connectors experience significant deformation, the shear capacity of the stud is primarily determined by the cross-sectional area at the stud’s root and yield strength. The impact of the stud’s length is minimal. Adopt the American code [66] and introduce partial factor ($k_{4}$). The Equation used to compute the effect of stud can be expressed as follows:

(17) $$P_{s}=n_{2}A_{s}f_{\mathrm{ys}}$$

where $n_{2}$ is the number of studs, $A_{s}$ is the cross-sectional area of studs and $f_{\mathrm{ys}}$ is the yield strength of the stud.

During loading, the stress state of the concrete varies in each hole of multi-hole shear connectors. Often, a portion of the concrete dowel is destroyed first, leading to a redistribution of structural stress. Only then did the other concrete dowel experience failure. Thus, the shear capacity of multi-hole connectors exhibits a notable decrease compared to the superimposition of the shear capacity of single-hole connectors [3]. The influence factor (λ) is introduced and calculated using the following Equation:

(18) $$\lambda =\frac{n_{1}d_{d}}{\left(n_{1}-1\right)l}$$

where l indicates the distance between neighboring holes.

The undetermined coefficients were determined by the use of the regression analysis methods, using the experimental data as a basis, where μ=1.25, $k_{1}=1.56$, $k_{2}=4.81$, $k_{3}=1.63$, $k_{4}=1.17$. Therefore, the shear capacity of the composite shear connectors can be computed as follows:

(19) $$P_{u}=1.56n_{1}\mu \lambda A_{r}f_{\mathrm{yr}}+4.81A_{c}f_{c}+1.63n_{1}\lambda A_{c}{f_{c}}^{0.57}+1.17n_{2}A_{s}f_{\mathrm{ys}}$$

The calculation formula had a clear physical meaning. The results obtained from these calculations are listed in Table 9.

**Figure 28:**
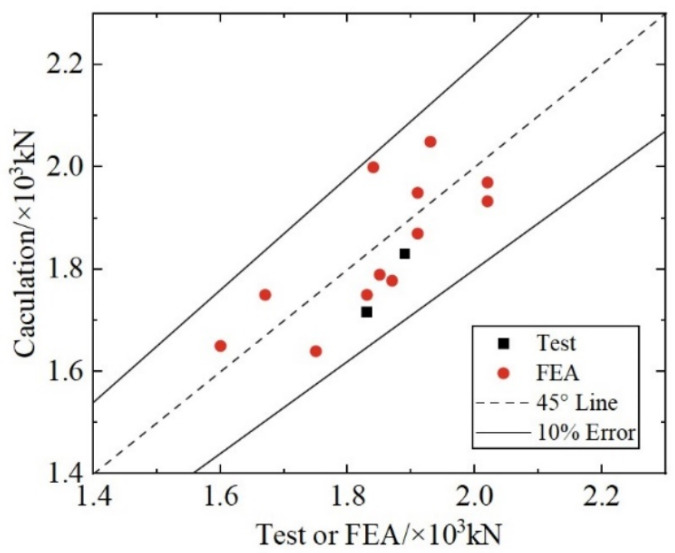
Evaluation of the Equation for composite shear connectors.

Figure 28 compares the test and FEA results with the calculated values obtained from Equation (19). All calculation errors are within 10%. It indicates that the results obtained from Equation (19) agree well with the test and FEA results.

## 6. Conclusions

This study envisaged a novel composite shear connector for the application of steel-concrete composite structures subjected to severe environmental actions. The experimental study, finite element analysis, and shear capacity predictions were carried out for the study of its load-carrying mechanisms. The following conclusions were drawn:

1. The composite shear connector had excellent shear resistance performance. Compared with traditional composite shear connectors, the shear capacity of SFCB-reinforced composite shear connectors was not far apart. However, there was a significant increase in shear stiffness and ductility of 7.85% and 63.61%, respectively. The application of SFCB elevated the shear stiffness. Simultaneously, it further enhanced the overall deformation capability of the structure, preventing concrete from splitting failure. It is believed that SFCB can be used in shear connectors to resist shear loads;
2. In the composite shear connectors, the load was shared by the studs, concrete dowels, penetrating rebars, and end-bearing concrete. The yielding of the penetrating rebar caused structural failure. The specimens exhibited good ductile behavior. It was found that the tensile fibers of SFCB were not destroyed after significant deformation and could provide stable “secondary stiffness” for the structure;
3. The finite element model that clearly demonstrated structural force transfer modes and damage forms was well fitted to the test results. The finite element analysis found that the number of holes impacting the structural shear capacity was not significant. However, the stud length, the diameter of the holes, and the CFRP thickness ratio had a big impact on the composite shear connectors. Therefore, great importance should be attached to the design of these parameters in applications;
4. A theoretical calculation model for the shear capacity of composite shear connectors was established. The results demonstrated good agreement between the calculated and experimental values, providing a valuable reference for engineering applications.

Future work includes the following:

1. Limited by the number of experimental specimens in this paper and due to the fact that the results of the numerical simulations tend to be idealized, experimental studies on the length of the pegs, the diameter and number of penetrations, and the thickness of the CFRP could be carried out if possible;
2. Research and development of finite element bonding unit for bonding between CFRP layer and concrete on the surface of SFCB;
3. If conditions permit, the novel composite shear connectors can be applied to composite structures and study their durability and seismic performance.

## Figures and Tables

**Table 1:** Group of push-out test specimens.

Specimen	Perforated Steel Plate				Penetrating Rebar		Stud		
	Height	Thickness	Hole Diameter	Hole	Diameter	Type	Length	Diameter	Number
	/mm	/mm	/mm	Number	/mm		/mm	/mm	
C-1-2	80	16	50	1	14	Steel bar	80	16	2 × 2
C-1-2-B	80	16	50	1	14	SFCB	80	16	2 × 2

**Table 2:** Mechanical parameters of concrete.

Material Type	Cube Compressive Strength	Elastic Modulus	Axial Compressive Strength	Cylindrical Compressive Strength
	(MPa)	(GPa)	(MPa)	(MPa)
C50	52.4	34.26	35.7	37.4

**Table 3:** Mechanical parameters of steel.

Material Type	Diameter	Yield Strength	Ultimate Tensile Strength	Elastic Modulus
	(mm)	(MPa)	(MPa)	(GPa)
HRB400	8	554.4	697.8	206.0
HRB400	14	483.6	677.2	206.0
Q235	16	363.1	442.5	206.0
Q355	-	375.3	501.4	206.0
SFCB	14	321.9	817.3	139.0

**Table 4:** Experiment results.

Specimen Number	$P_{u}$	$P_{\mathrm{rk}}$	$\delta_{u}$	$\delta_{0.9+}$	$\delta_{0.9-}$	$D_{c}$	*K*	Failure Member
	(kN)	(kN)	(mm)	(mm)	(mm)	$(\delta_{0.9u-}$$/\delta_{0.9u+}$)	(kN/mm)	
C-1-2	1887.87	1699.08	3.82	1.64	6.63	4.04	3440.67	Concrete
C-1-2-B	1827.39	1644.65	3.41	1.07	7.04	6.61	3711.23	SFCB

Note: The shear stiffness (K) is the secant slope value that corresponds to the load–slip curve of the specimen when it reaches a slip value of 0.2 mm. The ultimate shear capacity ($P_{u}$) is the largest shear force that could be achieved by the specimen over the whole of the loading process. The characteristic value of shear capacity ($P_{\mathrm{rk}}$), complies with the Eurocode 4, where $P_{\mathrm{rk}}=0.9P_{u}$. The peak slip ($\delta_{u}$) represents the amount of slip between the concrete slab and the H-beam when the ultimate shear capacity is reached. The slip values indicated as ($\delta_{0.9+}$) and ($\delta_{0.9-}$) the slip values associated with the characteristic shear capacity values in the ascending and descending portions of the load–slip curve, respectively. The ductility coefficient ($D_{c}$) is the ratio of the slip characteristic value ($\delta_{0.9-}$) to ($\delta_{0.9+}$).

**Table 5:** Parameters of cohesive element [50,51,52].

$\tau_{1c}$	$\tau_{2c}$	$\tau_{3c}$	$G_{1c}$	$G_{2c}$	$G_{3c}$	B-K Law Coefficient
(MPa)	(MPa)	(MPa)	(N/mm)	(N/mm)	(N/mm)	
57	90	90	0.28	0.63	0.63	1.8

**Table 6:** Parameters of CDP model [8,53].

Dilation Angle (ψ)	Eccentricity	$f_{b0}/f_{c0}$	*K*	$\mathrm{Viscosity}\;\mathrm{Parameter}\;(\mu_{v}$)
40	0.1	1.16	0.67	0.001

Notes: Typical values of ψ = 40 and Eccentricity = 0.1 are based on the recommendations of several studies [8,53]. The dilation angle is measured in the p–q plane at high confining pressure. In the p-q plane, p stands for the hydrostatic pressure stress, and q stands for the Mises equivalent effective stress. [54]; $f_{b0}/f_{c0}$ is stress ratio where the words mean the biaxial compressive strength of concrete and the uniaxial compressive strength of concrete, respectively, and *K* is a shape factor.

**Table 7:** Simulation parameters of CFRP [61,62,63,64].

Engineering Constants		Hashin Damage Parameters	
$E_{1}$/GPa	105.5	$X_{T}$/MPa	1340
$E_{2}$/GPa	7.2	$X_{C}$/MPa	1192
$E_{3}$/GPa	7.2	$Y_{T}$/MPa	19.6
$G_{12}$/GPa	3.4	$Y_{C}$/MPa	92.3
$G_{13}$/GPa	3.4	$S_{L}$/MPa	51
$G_{23}$/GPa	2.52	$S_{T}$/MPa	23
$\nu_{12}$	0.34	$G_{\mathrm{XT}}$/(N/mm)	48.4
$\nu_{13}$	0.34	$G_{\mathrm{XC}}$/(N/mm)	60.3
$\nu_{23}$	0.38	$G_{\mathrm{YT}}$/(N/mm)	4.5
-	-	$G_{\mathrm{YC}}$/(N/mm)	8.5

Notes: $E_{1}$, $E_{2}$ and $E_{3}$ are the elastic moduli along the three axes of the spatial coordinate system, respectively; $G_{12}$, $G_{13}$, $G_{23}$ are the shear moduli of the three planes in the spatial coordinate system, respectively; $\nu_{12}$, $\nu_{13}$, $\nu_{23}$ are the Poisson’s ratios of the three planes in the spatial coordinate system, respectively; $X_{T}$ and $X_{C}$ are longitudinal tensile and compressive strength, respectively; $Y_{T}$ and $Y_{C}$ are transverse tensile and compressive strength, respectively; $S_{L}$ and $S_{T}$ are longitudinal and transverse shear strength, respectively; $G_{\mathrm{XT}}$ and $G_{\mathrm{XC}}$ are longitudinal tensile and compressive fracture energy, respectively; $G_{\mathrm{YT}}$ and $G_{\mathrm{YC}}$ are transverse tensile and compressive fracture energy, respectively.

**Table 8:** Calculation comparison between the FEA model and test results.

Specimen	FEA Model Results/kN	Test Results/kN	Error
C-1-2	1751.15	1887.87	7.22%
C-1-2-B	1868.98	1827.39	2.28%

**Table 9:** Comparison between calculated and experimental values.

Specimens	$P_{\mathrm{Cal}.}$/kN	$P_{\mathrm{Exp}.}$/kN	$P_{\mathrm{Cal}.}$ $/P_{\mathrm{Exp}.}$
C-1-2	1751.01	1887.87	0.93
C-1-2-B	1716.98	1827.39	0.94
Mean	\	\	0.94
Standard deviation	\	\	0.005

## Data Availability

The original contributions presented in the study are included in the article, further inquiries can be directed to the corresponding author.
